# Simulation of adult limb regeneration with lizard tail spinal cord implants reveals distinct roles of radial glia and microglia populations

**DOI:** 10.21203/rs.3.rs-6010337/v1

**Published:** 2025-03-05

**Authors:** Ricardo Londono, Zheyu Pan, Megan L Hudnall, Thomas P Lozito

**Affiliations:** 1 Center for Cellular and Molecular Engineering, Department of Orthopaedic Surgery, University of Pittsburgh School of Medicine, Pittsburgh, PA, USA; 2 Department of Orthopaedic Surgery, Keck School of Medicine, University of Southern California, 1540 Alcazar St, Los Angeles, CA 90089, USA.; 3 Department of Stem Cell Biology and Regenerative Medicine, Keck School of Medicine, University of Southern California, 1425 San Pablo St, Los Angeles, CA 90033, US

## Abstract

Lizards are the closest relatives of humans able to suppress fibrosis and regrow multiple tissue lineages following appendage regeneration. As amniotes capable of tail, but not limb regrowth, lizards are also distinguished as the only vertebrate group that include both regenerative and non-regenerative appendages in the same animal. Lizard tail stumps naturally form blastemas - heterogenous collections of fibroblasts, adult stem cells, and immune cells that suppress scar formation and potentiate new tissue growth. Conversely, amputated lizard limbs form scars similar to those observed in human patients. Lizard blastema formation is dependent upon tail spinal cord tissue, which contains distinct populations of radial glia and microglia. Using the parthenogenetic lizard *Lepidodactylus lugubris* as a platform for tail-to-limb spinal cord implantations, we developed an ectopic blastema model toward defining the roles of radial glial and microglia in appendage regeneration. Removal of either population inhibits fibroblast proliferation and blastema formation, but only microglia depletion leads to enhanced fibrosis. Similarly, effects of radial glia, but not microglia, depletion on fibroblast proliferation are reversed via Hedgehog agonism. Taken together, these results indicate that lizard limbs contain all the necessary cell types and biological responses necessary for blastema formation but lack the proliferative and anti-fibrotic signals provided by tail spinal cord radial glia and microglia, respectively. Radial glia contribute Hedgehog signals that cause fibroblast proliferation but do not affect fibrosis. Conversely, microglia enhance fibroblast sensitivity to Hedgehog signaling and inhibit differentiation into fibrocytes. In summary, this study demonstrates blastema stimulation in amputated limbs of adult amniotes with application of lizard spinal cord cells and holds promise as a blueprint for limiting painful scarring and supporting new tissue growth following amputation injuries in human patients.

## Introduction

There are nearly 2 million people currently living with limb loss in the United States, and approximately 185,000 amputations occur each year [1]. Humans, like most mammals, suffer from minimal natural appendage regenerative capabilities. The ability to actually improve regenerative responses of patients and functionally regrow a complex structure like an arm is beyond the current limits of medical science. Remarkably, many animal species are naturally capable of these exact feats of wound healing [2–15]. The most widely studied of these “hyper-regenerative” organisms are the urodeles (newts and salamanders), which are capable of regrowing entire limbs and tails following amputation [6, 8–10, 16–20]. However, important differences between human and amphibian biology create barriers for translating salamander regenerative mechanisms to clinical relevancy. Indeed, urodeles exhibit larval developmental stages, cell types, and genetics that impart amazing regenerative processes without analogs in humans and other amniotes [21–28]. In fact, the urodele species most commonly used in regeneration studies, the axolotl salamander, exhibits neoteny, typically never metamorphosing from its larval form. The absence of larvae in amniotes makes meaningful comparisons with salamanders difficult. Among mammals, the best characterized model for regeneration of limb structures is the mouse digit tip [5]. However, this model suffers from size and lineage restrictions; only a small amount of bone under the nail bed is regrown, and other non-osseous tissues such as fat, cartilage, and muscle are not naturally regenerated.

Lizards are the closest relatives of mammals and the only adult amniotes capable of multi-lineage appendage regeneration. Hence, lizards represent an informative model organism to bridge this gap [11, 12]. For example, lizards follow a similar developmental plan as mammals yet are capable of tail regrowth as adults, making lizard regeneration particularly relevant from biological and developmental standpoints. Lizards and mammals also share similarly advanced immune systems and inflammatory responses to injury, highlighting lizards’ particular applicability to the study of appendage regeneration amidst complex wound environments [29]. Finally, while many lizard species are able to regrow lost tails, no lizard is able to naturally regenerate amputated limbs [12, 30], distinguishing lizards as the only adult tetrapods that combine regenerative and non-regenerative appendages in the same animal [12, 30, 31]. While amputated lizard tails form blastemas, specialized regenerative structures that generate new tissues, lizard limbs scar over in a manner similar to mammalian amputation injury responses [32]. The strikingly divergent regenerative outcomes exhibited by lizard limbs versus tails present compelling opportunities for comparative studies aimed at identifying the cellular and molecular determinants of scar inhibition, blastema formation and regeneration. This study attempts to answer the question “Why can lizards regenerate tails but not limbs?”.

Previous studies have reported on the nerve-dependence of appendage regeneration in a wide range of injury models and species [25, 33–40], including nerve depletion studies in urodeles. Here, nerves are severed prior to limb amputation, resulting in loss of blastema formation and regeneration in newts and salamanders [33]. Subsequent studies concluded that nerves contribute secreted factors, including substance P [41, 42], insulin [43], transferrin [44, 45], nerve growth factor [46, 47], and fibroblast growth factors [48, 49], that increase limb blastema cell proliferation and suppress fibrosis. Nerve-dependency of amphibian tail regeneration has also been demonstrated, but instead of peripheral nerves, tail regrowth is regulated by spinal cord tissue [50, 51]. Importantly, in both limb and tail models, nerve-support cells, such as glia and fibroblasts, are also reported to contribute to the pro-regenerative activities of nervous tissues [25, 52–55]. These support cells may be particularly relevant in lizard blastema formation, since lizards do not regenerate spinal cord neurons during tail regrowth. For example, lizard radial glial cells (RGCs), not neurons, regulate lizard blastema cell differentiation via Hedgehog (HH) signaling [30, 56, 57].

A separate set of studies have identified phagocytes as regulators of wound healing in a wide range of animal models, including lizards [29, 30, 58–60]. Specifically, phagocytes were shown to migrate to wound sites, where they contribute signaling molecules, down-regulate pro-inflammatory cytokines, reduce levels of reactive oxygen species, remodel extracellular matrix, induce wound re-epithelization, promote tissue histolysis, and support blastema formation [61]. Many of the experiments used in these studies employed systemic clodronate liposome treatments to nonspecifically deplete phagocytes populations [30, 58, 60] and could not distinguish among effects contributed by macrophages, osteoclasts, neutrophils, mast cells, monocytes, tissue dendritic cells, and microglia. Of these phagocyte populations, only microglia are found in lizard tails but not limbs [30], marking these cells as particularly attractive candidates for active participants blastema formation.

In this study, we investigate the roles of spinal cord radial glia and microglia in lizard blastema formation. In order to overcome challenges associated with specifically targeting microglia, we developed an ectopic blastema model capable of inducing limb regeneration through the application of lizard tail spinal cord implants.

## Materials and Methods

All reagents/chemicals were purchased from Sigma-Aldrich unless otherwise specified. All experiments complied with relevant ethical regulations for animal testing and research.

### Lizard husbandry and species selection

Green anole lizards (*Anolis carolinensis*) were maintained on 12-h light/12-h dark schedules with 100 W UVA/UVB heat lamps. Anoles were housed in metal mesh cages at 65% humidity and temperatures of 24–26 °C during light hours and 18.5–21 °C during dark hours. Water was provided by misting five times per week, and lizards were fed ½-inch crickets dusted in calcium supplement three times per week. Male and female anoles, ages 9–12 months old, were tested equally in all experiments.

Ectopic blastema experiments were carried out using mourning geckos (*Lepidodactylus lugubris*). This parthenogenetic lizard species reproduces asexually, yielding clonally identical offspring [62]. Cells and tissues can be transplanted between colony members in the absence of immunosuppressants and anti-rejection therapeutics [30, 63], which have been shown to impact tail regeneration [60, 64, 65]. Mourning geckos were maintained at 24–26 °C during light hours and 18.5–21 °C during dark hours in plastic cages, with 65% humidity, water misting three times per week, and were fed a diet of fruit meal replacement powder 3 times per week. All mourning geckos utilized in experiments were ages 9–12 months old. Husbandry and experimental use of lizards were conducted per guidelines of the Institutional Animal Care and Use Committees at the University of Pittsburgh (protocols 15114947, 16128889, and 18011476) and the University of Southern California (protocols 20992 and 21148).

### Lizard tail and limb amputations

Lizard tail and limb tissue collection involves 2 amputations per animal. First amputations removed original tails/limbs and created wounds. Lizard tails were anesthetized with 10 second ethyl chloride sprays and amputated with sterile scalpel blades halfway along tail lengths to begin regeneration.

During lizard limb amputation procedures, lizards were anesthetized via intramuscular injections of Alfaxan (10–50 mg/kg body weight). Full anesthetization was determined by lack of movement and lack of response to stimuli (pinching the tail with forceps). Subcutaneous bupivacaine / epinephrine (1 mg/kg) was administered once prior to surgical incisions for analgesia and to assist with hemostasis. Right hind limbs were sterilized with alternating scrubs of betadine and alcohol and amputated with sterile scalpel blades halfway between knees and ankles. Tibia and fibula bones were trimmed so that they did not interfere with re-epithelialization. A single dose of meloxicam (0.2 mg/kg) was administered via subcutaneous injection as analgesia. Lizards were carefully monitored until they were alert and active.

To collect tissues for histological analysis and cell isolations, lizards were euthanized, and second amputations were performed 5 mm distal to original amputation planes typically14 or 21 days post-amputation (DPA). Tail and limb samples were collected in Hank’s balanced salt solution (HBSS) supplemented with 100 units/mL penicillin and 100 μg/mL streptomycin (HBSS with P/S). Lizard euthanasia was performed in a manner consistent with the recommendations of the American Veterinary Medical Association (AVMA) Guidelines according to the protocol: (1) inject 25–50 μl of pH-neutralized 1% MS222 into the coelom. (2) after loss of righting reflex, inject 25–50 μl 50% (v/v) MS222 into the coelom. (3) decapitation. (4) double pithing brains/spinal cords.

### Ectopic Limb Blastema Model

First, regenerated spinal cords were isolated according to published protocols [56]. Briefly, regenerated *L. lugubris* gecko tails were collected 21 DPA and cut into 5 mm sections. Tail sections were cut along dorsal and ventral midlines down to the depth of cartilage tubes. Skin, muscle, and connective tissue were laterally peeled to expose cartilage tubes. In mature regenerates, there exists very little connection between cartilage tubes and non-skeletal tissues, and cartilage tubes are easily separated from other tail tissues. To isolate regenerated spinal cords, cartilage tubes were cut along dorsal and ventral midlines to the depth of inner tube edges. Cartilage tubes were separated along cut lines. Very few tissue connections exist between spinal cords and cartilage tubes, and intact lengths of regenerated spinal cords are easily isolated from remaining cartilage tissue. Next, legs of recipient geckos *L. Lugubris* were amputated as described above. Finally, isolated regenerated tail spinal cords were inserted into amputated recipient lizard limbs into areas left from trimmed tibia or fibula.

### Drug treatments

For HH signaling modulation, lizards were treated with cyclopamine (25 μg drug/g animal), or smoothened agonist (SAG, 20 μg/g) via intraperitoneal (IP) injections every 48 h. Control animals were treated with vehicle controls. For experiments involving diphtheria toxin (DT) treatment, lizards received IP injections of 4 ng DT/g animal or vehicle control (PBS). For labeling proliferating cells, lizards were treated with 100 μg/g 5-ethynyl-2’-deoxyuridine (EdU) 4 hours prior to sample collections as previously described [28].

### Virus treatments

RGC labeling was carried out using AAV5 packaged with pAAV.GFAP.eGFP.WPRE.GH (GFAPp-eGFP). pAAV.GFAP.eGFP.WPRE.GH was a gift from James M. Wilson (Addgene viral prep #105549-AAV5; http://n2t.net/addgene:105549; RRID: Addgene_105549). RGC depletion was carried out using combined treatments of AAV5 packaged with pAAV5.GFAP.Cre.WPRE.hGH (GFAPp-Cre) and AAV5 packaged with pAAV5-FLEX-DTR-GFP (FLEX-DTR-GFP). pAAV5.GFAP.Cre.WPRE.hGH was a gift from James M. Wilson (Addgene viral prep #105550-AAV5; http://n2t.net/addgene:105550; RRID: Addgene_105550). pAAV5-FLEX-DTR-GFP was a gift from Eiman Azim and Thomas Jessel (Addgene viral prep #124364-AAV5; http://n2t.net/addgene; RRID: Addgene_124364). 1.0×10^10^ viral particles of each type were delivered to central canals of amputated tail stumps using a custom microinjection system.

### Liposome treatment

For phagocyte depletion, lizards were treated with IP injections of l-α-phosphatidylcholine/cholesterol liposomes containing clodronate (0.125 mg/g animal weight) 72 and 48 h prior to amputation. Control animals were treated with liposomes containing PBS instead of clodronate. To fluorescently label phagocyte populations, 1,1′-dioctadecyl-3,3,3′,3′-tetramethylindocarbocyanine (DiI) liposomes were delivered via IP injections (0.125 mg/g animal weight), typically 4 hours prior to sample collections.

### Histology and Imaging

Lizard tail and limb samples were fixed, decalcified, equilibrated as previously described [30]. Samples were then equilibrated via sucrose gradients before being snap frozen in Optimal Cutting Temperature Compound (OCT) via 2-methylbutane / dry ice baths. 16 μm cryosections were collected using a Leica CM1860 cryostat. All images of sagittal sections are presented in figures with dorsal tail toward the top, ventral toward the bottom, distal toward the right, and proximal toward the left, unless otherwise noted. Processed samples were imaged with a Keyence BZ-X810 Microscope as previously described [30]. Z-stacks and stitched images were processed using BZ-X800 Analyzer software (Keyence, v1.1.1.8), according to the manufacturer’s instructions. Adobe Photoshop 2024 and Illustrator 2024 were used for preparing images and figures for publication.

### In situ hybridization and immunofluorescence

Fluorescent in situ hybridization (FISH) was performed using the RNAscope^™^ Multiplex Fluorescent Reagent Kit v2 (Advanced Cell Diagnostics) and custom proprietary FISH probes (Supplementary Table 1). FISH was carried out according to protocols validated for lizard tissue samples [30].

Immunofluorescence (IF) was performed as previously described [56] using primary antibody for GFAP (Abcam ab4674, 1:1000).

### Image acquisition and processing

Processed samples were imaged with a Keyence BZ-X810 Microscope as previously described [30]. Z-stacks and stitched images were processed using BZ-X800 Analyzer software (Keyence, v1.1.1.8), according to the manufacturer’s instructions. Adobe Photoshop 2024 and Illustrator 2024 were used for preparing images and figures for publication. All images of sagittal sections are presented dorsal toward the top, ventral toward the bottom, distal toward the right, and proximal toward the left. Transverse sections are presented with dorsal on top and ventral on bottom. Positive FISH staining areas were quantified using Fiji (Image J, NIH) applying over/under thresholding to limit analysis to positive staining areas.

### Statistics and reproducibility

Statistical analysis was performed using Prism 7 with one- or two-way ANOVA with pairwise Tukey’s multiple comparison test for data with multiple groups. A p value of <0.05 was deemed to be statistically significant. All values and graphs are shown as mean ± SD. Each experiment used to generate micrographs were independently repeated at least five times, and micrographs of representative samples are presented.

## Results

### Regenerated lizard tail spinal cords contain populations of RGCs and microglia.

**Figure 1:**
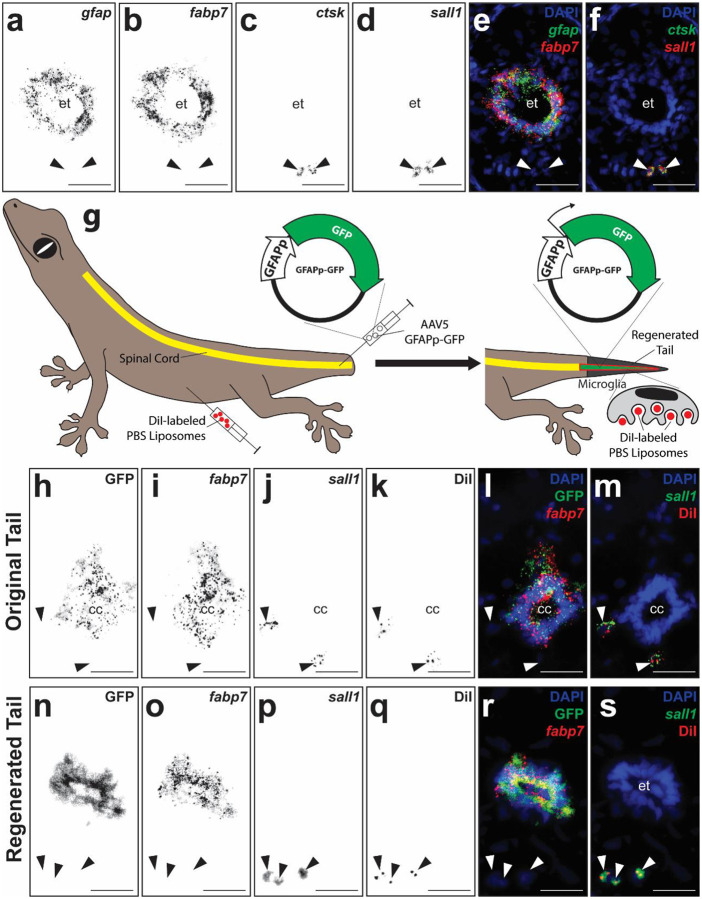
RGCs and microglia are incorporated into regenerated lizard tail spinal cords. **(a-f)** Representative transverse sections of regenerated lizard tail spinal cords (21 DPA) analyzed by IF/FISH for *gfap*, *fabp7*, *ctsk*, and *sall1* expression. **(g)** Experimental scheme for labeling and lineage tracing RGC and phagocyte populations during lizard tail regeneration. RGCs and phagocytes within original tails were pre-labeled with AAV5 (GFAPp-GFP) and DiI liposomes, respectively. Following tail amputations and 21 days of regrowth, regenerated tails were collected and analyzed for co-localization of GFP and DiI signals with RGC and/or microglia markers. **(h-s)** Representative sagittal sections of (h-m) original and (n-s) regenerated tails collected from lizards treated with AAV5(GFAPp-GFP) and DiI liposomes and analyzed by histology/FISH for GFP and DiI fluorescence signals and *gfap*, *fabp7*, *ctsk*, and *sall1* expression. Arrow heads mark DiI^+^ *sall1*^+^ microglia. et, ependymal tube. Bar = 50 μm.

We have previously demonstrated the importance of *gfap*^+^ *fabp*7^+^ RGCs and *ctsk*^+^ phagocytes in lizard tail regeneration [28–30, 56, 63]. Here we confirmed their presence in regenerated lizard tail spinal cord tissue and tested methodology for labeling their populations in vivo. Cross sections of regenerated lizard tails (21 DPA) were analyzed by histology/immunofluorescence (IF)/fluorescent in situ hybridization (FISH) for *gfap*, *fabp7*, and *ctsk* expression (Fig. 1 a–f). Expression of microglia marker *sall1* [66, 67] was also assayed by ISH to distinguish microglia from other spinal cord phagocyte populations (Fig. 1 a, f). *Gfap*^+^ *fabp7*^+^ RGCs were detected among ependymal cell populations lining central canals of ependymal tubes, while *ctsk*^+^ phagocytes were detected in regenerated spinal cord tissue immediately surrounding ependymal tubes (within 150 μm) (Fig. 1 a–f). The vast majority (over 98%) of regenerated spinal cord phagocytes, but not other tail phagocytes, co-expressed *sall1*, indicating a microglia identity (Fig. 1 f, Supplementary Figure 1). RGC and microglia marker expression did not overlap, confirming these two cell populations as distinct (Supplementary Figure 2).

To label RGCs in vivo, we took advantage of a system previously validated for labeling ependymal cells in regenerating salamander tails [18]. RGCs within amputated tail stumps were transduced with AAV5 containing an expression construct containing GFP driven by a highly conserved region of the GFAP promoter (GFAPp-GFP). Viral vectors were delivered to central canals of spinal cords in amputated tail stumps via microinjection (Fig. 1 g). Following 21 days, tails were collected and analyzed by histology/ISH for GFP and *fabp7* expression (n=5). In transversely sectioned samples, GFP and *fabp7* co-localized in RGC populations of both original and regenerated tail ependyma (Fig. 1 h–s). In samples sagittally sectioned through junctions between original and regenerated tail regions, *gfap*^+^ *fabp7*^+^ RGC populations within ependyma of original tail spinal cords were continuous with those of regenerated ependymal tubes (Supplementary Figure 3). Taken together, these results suggested that RGCs labeled at amputation sites contribute to ependymal tubes in regenerated tails.

Lizard phagocytes were specifically labeled with DiI-containing PBS liposomes as previously described [29]. Lizards were pre-treated with DiI liposomes 48 hours prior to tail amputation (Fig. 1 g). Following 21 days of regrowth, lizard tails were analyzed by histology/IF for *sall1* expression and DiI signal. Transverse sections of both original and regenerated tail regions exhibited DiI^+^ *sall1*^+^ microglia (Fig. 1 h–s), indicating that, like RGCs, microglia within regenerated tail spinal cords originated originate from original tail populations. Furthermore, DiI/*sall1* and GFP/*fabp7* signals did not co-localize in either original or regenerated tail regions (Fig. 1 h–s), suggesting that AAV5 (GFAPp-GFP) and DiI liposomes labeled distinct populations of RGC and microglia, respectively.

### RGCs are required for blastema fibroblast proliferation but not fibrosis suppression.

**Figure 2:**
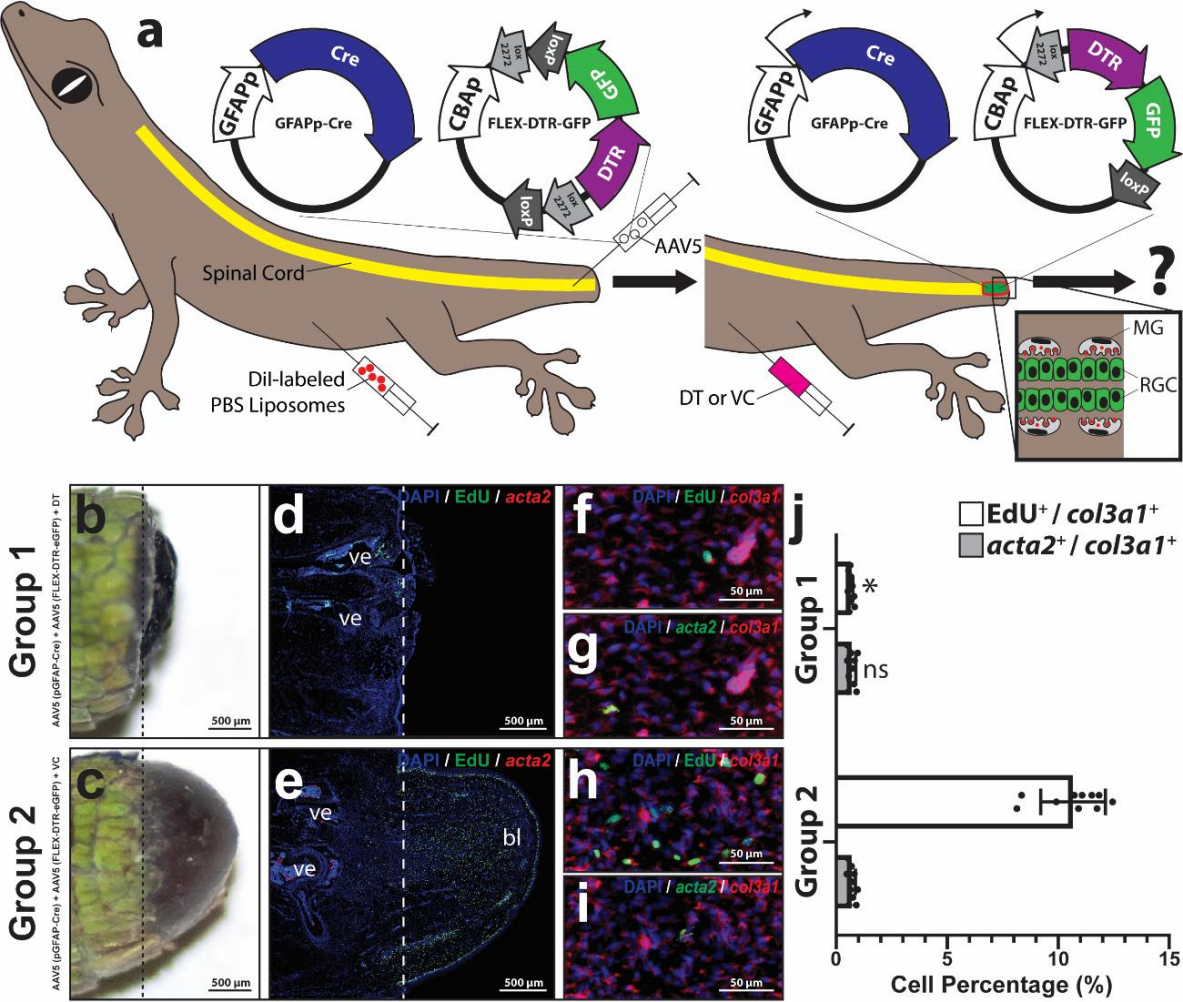
Lizard spinal cord RGC depletion inhibits tail fibroblast proliferation but does not affect fibrosis. (a) Experimental scheme used to deplete RGCs from spinal cords of amputated lizard tails. RGCs within original tails were pre-labeled with AAV5 (GFAPp-Cre) and AAV5 (FLEX-DTR-GFP). In the presence of Cre, the FLEX-DTR-GFP construct recombines to allow expression of diptheriatoxin receptor (DTR) and GFP off the same chicken β-actin promoter (CBAp). Lizard were also treated with DiI liposomes to label phagocytes. 7 DPA, lizards were treated with diphtheria toxin (DT) or vehicle control (VC). At 14 DPA, tail samples were collected and analyzed for effects on blastema formation, including fibroblast proliferation and fibrocyte differentiation. **(b, c)** Gross morphology of representative tails collected 14 DPA from (b) Group 1 and (c) Group 2 lizards. **(d, e)** Sagittal sections of Group 1 and 2 lizard tails analyzed 14 DPA by FISH and fluorescence microscopy for EdU incorporation (cell proliferation) and expression of *acta2* (fibrocyte/myofibroblast marker). Dashed lines mark amputation planes. **(f-i)** Higher magnification views of distal tail regions analyzed by histology/FISH for EdU incorporation and expression of *col3a1* (general fibroblast marker) and *acta2*. (j) Quantified percentages of *col3a1*^+^ fibroblasts from Group 1 or Group 2 tails that exhibit EdU incorporation or express *acta2* 14 DPA. n = 10 different animals/tails for each condition. Data are presented as mean values +/− SD. Two-way ANOVA with pairwise Tukey’s multiple comparison tests was used. *, p < 0.01; ns, not significant; compared to corresponding Group 2 values.

The effects of targeted depletion of RGCs on blastema development and scar formation following lizard tail amputation were studied (Fig. 2; Supplementary Figures 4–6). RGCs were depleted by combining GFAPp-Cre with a Cre-responsive diphtheria toxin receptor (DTR) system (FLEX-DTR-GFP). In the presence of Cre, *gfap*^+^ transduced cells and their progeny express a DTR-GFP fusion protein that, upon treatment with diphtheria toxin (DT), are selectively ablated (Fig. 2 a). First, we validated this system for labeling and ablating lizard spinal cord RGCs (Supplementary Figure 4). AAV5 (GFAPp-Cre) and AAV5 (FLEX-DTR-GFP) were micro-injected into spinal cord central canals of freshly amputated lizard tails. Negative control tails received micro-injections of AAV5 (FLEX-DTR-GFP) but not AAV5 (GFAPp-Cre). Following one week, lizards were systemically treated with either diphtheria toxin (DT) or vehicle control (VC). Thus, there were 4 experimental groups: (Group 1) AAV5 (GFAPp-Cre) + AAV5 (FLEX-DTR-GFP) + DT; (Group 2) AAV5 (GFAPp-Cre) + AAV5 (FLEX-DTR-GFP) + VC; (Group 3) AAV5 (FLEX-DTR-GFP)+ DT; (Group 4) AAV5 (FLEX-DTR-GFP) + VC. At 14 DPA, lizards were treated with DiI liposomes to label phagocytes, and tails were analyzed by gross morphology (Fig. 2 b, c; Supplementary Figure 5 a, b) and histology/FISH for *fabp7* and *sall1* expression and for GFP and DiI fluorescence signals (Supplementary Figure 4). All tails except those of Group 1 formed blastemas (Fig. 2 b, c; Supplementary Figure 5 a, b), and Group 1 tails exhibited significantly less new tissue distal to amputation planes (Supplementary Figure 6). RGC depletion was validated via assessments of *fabp7* FISH and GFP signals within spinal cords of control and experimental groups (Supplementary Figure 4 a-x). GFP signal was only observed in tails of Group 2 and co-localized with *fabp7* expression (Supplementary Figure 4 g-l), indicating efficient Cre-mediated labeling of RGCs via the FLEX system. Both GFP and *fabp7* signal were reduced in DT-treated Group 1 samples compared to Group 2 (Supplementary Figure a-f), indicating efficient ablation of RGCs by DTR/DT. DT treatment also destroyed ependymal tube structures within Group 1 blastemas (Supplementary Figure 4 a-f). Conversely, GFP expression was not observed in *sall1*^+^ DiI+ microglia (Supplementary Figure 4 g-l), and none of the treatments tested had significant effect on microglia numbers (Supplementary Figure 4 a-x). Taken together, these results validated the GFAPp-Cre/Flex-DTR-GFP system as a specific and efficient method for specifically labeling and depleting RGCs in regenerating lizard tails.

Finally, we analyzed the effects of RGC depletion on lizard fibroblast proliferation and scar formation (Fig. 2, Supplementary Figure 5). Tail samples from each group were analyzed for cell proliferation via EdU incorporation, and by FISH for lizard fibroblast marker *col3a1* [30] and fibrosis marker *acta2*. Negative control tails (Group 2) and VC tails (Groups 3 and 4) formed normal blastemas containing high levels of *col3a1*^+^ EdU^+^ proliferating fibroblasts and low levels of *col3a1*^+^ *acta2*^+^ fibroblasts (Fig. 2 e, h–j; Supplementary Figure 5 c-i). Conversely, RGC depletions via DT treatments (Group 1) significantly inhibited tail blastema formation and *col3a1*^+^ fibroblast proliferation (Fig. 2 b, d, f, j). Interestingly, RGC depletion (Group 1) did not affect *acta2* expression (Fig, 2 d, g, j) compared to control conditions (Group 2–4), indicating that RGCs regulate blastema cell proliferation but do not play roles in scar suppression.

### Phagocytes are required for both fibroblast proliferation and scar suppression during lizard tail regeneration.

**Figure 3:**
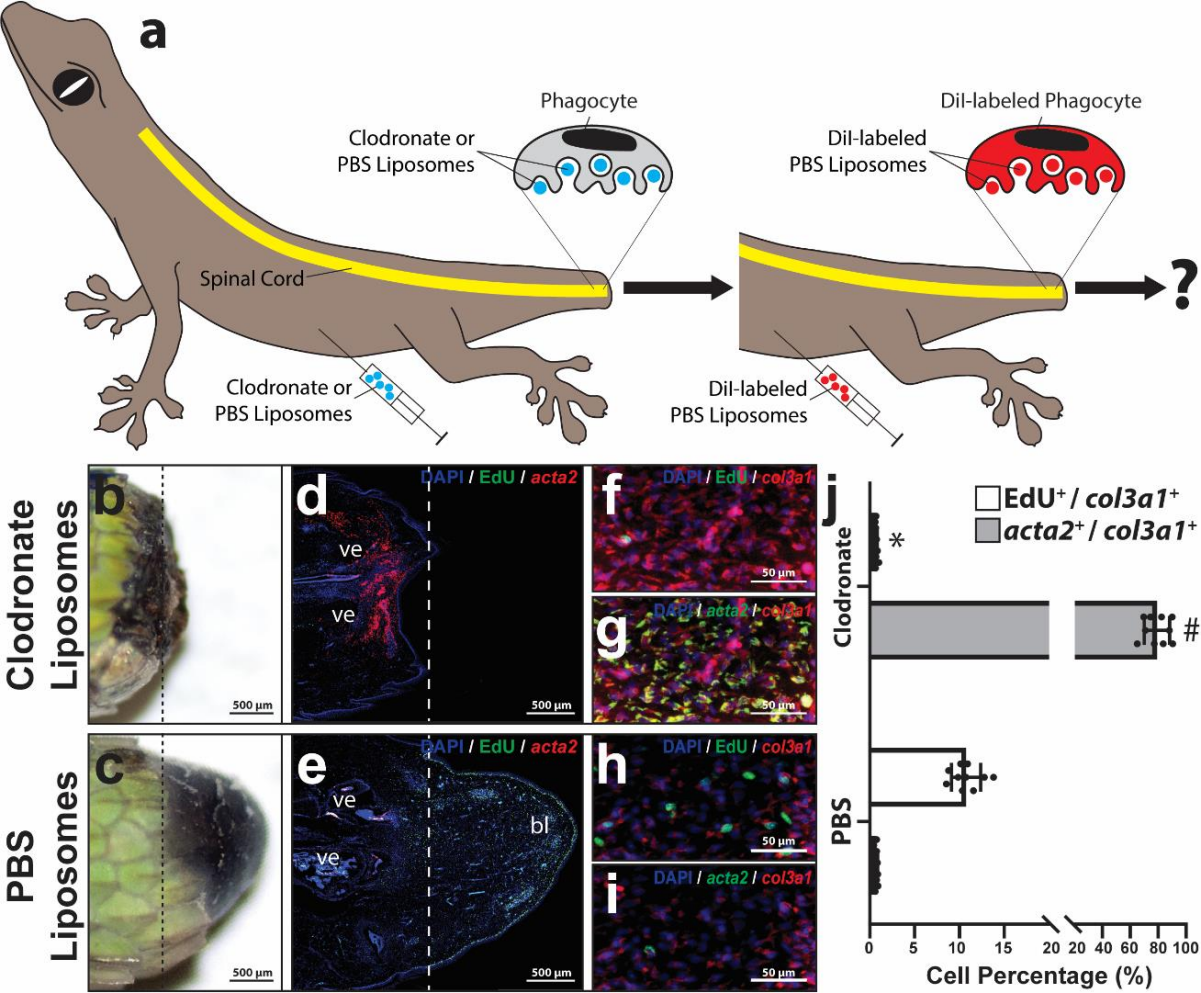
Phagocyte depletion inhibits both fibroblast proliferation and scar formation. **(a)** Experimental scheme used to systemically deplete phagocytes from lizards during amputated tail healing. Lizards were treated with clodronate or PBS (control liposomes) to modulate phagocyte levels. At 14 DPA, tail samples were collected and analyzed for effects on fibroblast proliferation and scar formation. **(b, c)** Gross morphology of representative tails collected 14 DPA from lizards treated with (b) clodronate and (c) PBS liposomes. **(d, e)** Sagittal tail sections collected from lizards treated with clodronate or PBS liposomes analyzed 14 DPA by FISH/fluorescence microscopy for EdU incorporation and acta2 expression. Dashed lines mark amputation planes. **(f-i)** Higher magnification views of distal tail regions analyzed by histology/FISH for EdU incorporation and expression of col3a1 and acta2. **(j)** Quantified percentages of col3a1^+^ tail fibroblasts that exhibit EdU incorporation or express acta2 following treatment with clodronate or PBS liposomes (14 DPA). n = 10 different animals/tails for each condition. Data are presented as mean values +/− SD. Two-way ANOVA with pairwise Tukey’s multiple comparison tests was used. *, # p < 0.01compared to corresponding PBS liposome (control) values.

The effects of phagocyte depletion on lizard blastema development and fibrosis following tail amputation were determined (Fig. 3, Supplementary Figure 7–8). Lizard were treated with clodronate liposomes 72 and 24 hours prior to tail amputation. Vehicle control lizards were treated with PBS liposomes. Experimental and control lizards (n=10 per condition) were treated with DiI liposomes 4 hours prior to collection to systemically label cells based on phagocytic activity, and tail samples were collected 14 DPA (Fig. 3 a) and analyzed via gross morphology and histology/FISH for *fabp7*, *sall1*, *col3a1*, *acta2*, and EdU expression (Fig. 3 a; Supplementary Figure 7, 8). Systemic phagocyte depletion in lizards using clodronate liposome treatments was previously demonstrated by our group [29], and here we validated that this method also depleted microglia populations of amputated tails (Supplementary Figure 7). Control tails of PBS liposome-treated lizards exhibited DiI^+^ *sall1*^+^ microglia associated with - but distinct from - *fabp7*^+^ RGCs within spinal cords (Supplementary Figure 7 a-e), while clodronate liposome treatments caused a disappearance of microglia at amputation planes without affecting RGCs (Supplementary Figure 7 f-j). These results confirm that clodronate liposome treatments deplete lizard spinal cord microglia following tail amputations.

Phagocyte depletion also significantly inhibited blastema formation and reduced fibroblast proliferation (Fig. 3 b, d, f, j; Supplementary Figure 8). In contrast to RGC depletion, chlodronate liposome treatments also significantly increased fibroblast *acta2* expression (Fig. 3 d, g, j). Taken together, these results suggest that phagocytes, but not RGCs, suppress fibrosis / fibrocyte differentiation during blastema formation. These experiments also highlighted the need for alternative strategies to specifically test the roles of spinal microglia in lizard blastema development.

### Hedgehog agonists rescue lizard tail regeneration following RGC, but not phagocyte, depletion.

**Figure 4:**
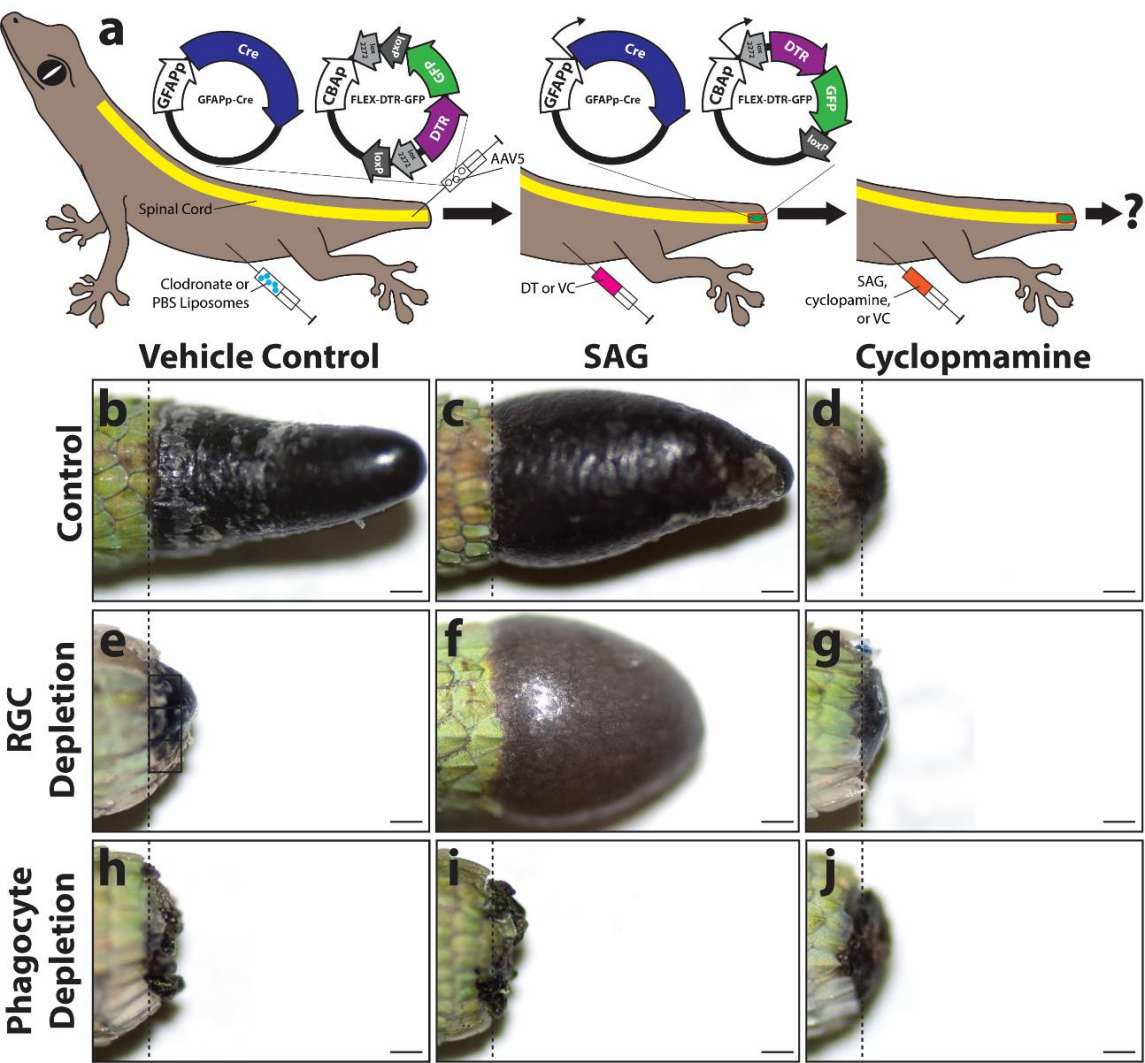
Effects of HH stimulation and inhibition on lizard tail regeneration following RGC or phagocyte depletion. (a) Experimental scheme used test abilities of HH agonist SAG and HH inhibitor cyclopamine to rescue lizard tail regeneration following RGC or phagocyte depletion. Lizard tail spinal cords were pre-transduced with AAV5(GFAPp-Cre) and AAV5(FLEX-DTR-GFP). RGCs were depleted by treating with DT. Phagocytes, including microglia, were depleted by treatment with clodronate liposomes. Lizards were treated with SAG, cyclopamine, or vehicle control. At 21 DPA, tails were collected and analyzed for effects on blastema formation and scar deposition. **(b-j)** Gross morphologies of (b-d) control tails, (e-g) RGC-depleted tails, and (h-j) tails of phagocyte-depleted lizards (21 DPA) treated with vehicle control, SAG, or cyclopamine. Dashed lines mark amputation planes. Bar = 500 μm.

We have previously identified Hedgehog (HH) signaling as the primary regulator of lizard blastema fibroblast proliferation and chondrogenesis and identified spinal cord RGCs as the endogenous source of sonic hedgehog (SHH) during lizard tail regeneration [28, 30, 56, 57, 63, 68]. Finally, we validated smoothened agonist (SAG) and smoothened inhibitor cyclopamine as effective modulators of lizard HH signaling in vivo [28, 30, 56, 63, 68]. Here we tested the abilities of HH stimululation via SAG treatments to rescue fibroblast proliferation and fibrosis suppression in the absence of either RGCs or phagocytes (Fig. 4, Supplementary Figure 9, 10). The following conditions were tested: (1) no cell depletion + vehicle control; (2) no cell depletion + SAG; (3) no cell depletion + cyclopamine; (4) RGC depletion + vehicle control; (5) RGC depletion + SAG; (6) RGC depletion + cyclopamine; (7) phagocyte depletion + vehicle control; (8) phagocyte depletion + SAG; phagocyte depletion +cyclopamine (Fig. 4; Supplementary Figure 9, 10). As described above, RGC depletion was accomplished via diphtheria toxin treatments in lizards pre-transduced with both GFAPp-Cre and Flex-DTR-GFP constructs (Fig. 4 a). Phagocytes were depleted by pre-exposure to clodronate liposomes (Fig. 4 a). Lizards were treated with SAG, cyclopamine, or vehicle control every 72 hours following tail amputations, and tails from all groups were collected 21 DPA (Fig. 4 a). Tail samples were analyzed by gross morphology (Fig. 4 b–j; Supplementary Figure 9) and by histology/ISH for EdU, *col3a1*, and *acta2* to assess fibroblast proliferation and fibrocyte differentiation (Supplementary Figure 10). Control tails with intact RGC and microglia populations regenerated normal tails. (Fig. 4 b; Supplementary Figure 9). Treatment with SAG significantly expanded regenerated area but not length (Fig. 4 c; Supplementary Figure 9), while cyclopamine treatment inhibited lizard tail regeneration (Fig. 4 d; Supplementary Figure 9). Similarly, fibroblast proliferation was significantly increased following SAG treatment and significantly decreased in response to cyclopamine treatment (Supplementary Figure 10). Both RGC and phagocyte depletion reduced fibroblast proliferation (Supplemenary Figure 10), and vehicle control treatments did not induce any growth past amputation planes in either RGC or phagocyte depletion conditions (Fig. 4 e, h; Supplementary Figure 9). SAG treatment induced significant, bulbous tail growth in RGC, but not phagocyte, depletion conditions (Fig. 4 f, I; Supplementary Figure 9) and rescued fibroblast proliferation following RGC, but not phagocyte, depletion (Supplementary Figure 10). Cyclopamine treatment did not affect fibroblast proliferation following with RGC or phagocyte depletions (Supplementary Figure 10).

Conversely, none of the tested HH-modulating treatments affected fibrocyte differentiation (Supplementary Figure 10). Elevated *acta2* expression levels were observed following phagocyte, but not RGC, depletion and were not affected by either SAG or cyclopamine treatments (Supplementary Figure 10). Taken together, these results suggest that RGCs regulate blastema fibroblast proliferation via HH signaling, but neither RGCs nor HH signaling regulate fibrocyte differentiation. Conversely, phagocytes act upstream of RGCs to support fibroblast responsiveness to HH signals and suppress fibrocyte differentiation in an HH-independent manner.

### Regenerated spinal cord implants induce ectopic limb blastema formation

Having demonstrated the importance of *gfap*^+^ *fabp7*^+^ and *ctsk*^+^ *sall1*^+^ microglia in tail regeneration, we sought to determine if similar endogenous cell populations are present in naturally non-regenerative lizard limbs. Tails and limbs collected from lizards treated with DiI liposomes were analyzed by histology/FISH 0, 7, 14, and 21 DPA (Supplementary Figure 11). Lizard limbs exhibited *gfap*^+^ *fabp7*^−^ glial cells, particularly at 7 DPA, but these cells did not significantly contribute to later healing stages (Supplementary Figure 11 a). In contrast to regenerating tails, amputated limbs lacked *gfap*^+^ *fabp7*^+^ RGCs (Supplementary Figure 11 a), as expected from the absence of endogenous CNS tissues. Maybe talk about ependymal tube contribution

Lizard limbs exhibited *ctsk*^+^ *sall1*^−^ phagocytes but lacked *ctsk*^+^ *sall1*^+^ microglia found in tails (Supplementary Figure 11 b). *Ctsk*^+^ *sall1*^−^ phagocytes followed similar dynamics in both limb and tail time course, peaking at 7 DPA before decreasing at 14 and 21 DPA (Supplementary Figure 11 b). Limbs lacked *ctsk*^+^ *sall1*^+^ microglia (Supplementary Figure 11 b), again reflective of the absence of exogenous CNS elements. Tail *ctsk*^+^ *sall1*^+^ peaked at 14 DPA blastema stages (Supplementary Figure 11 b). Taken together, these results highlighted RGCs and microglia as the specific spinal cord cell populations associated with blastema formation.

**Figure 5:**
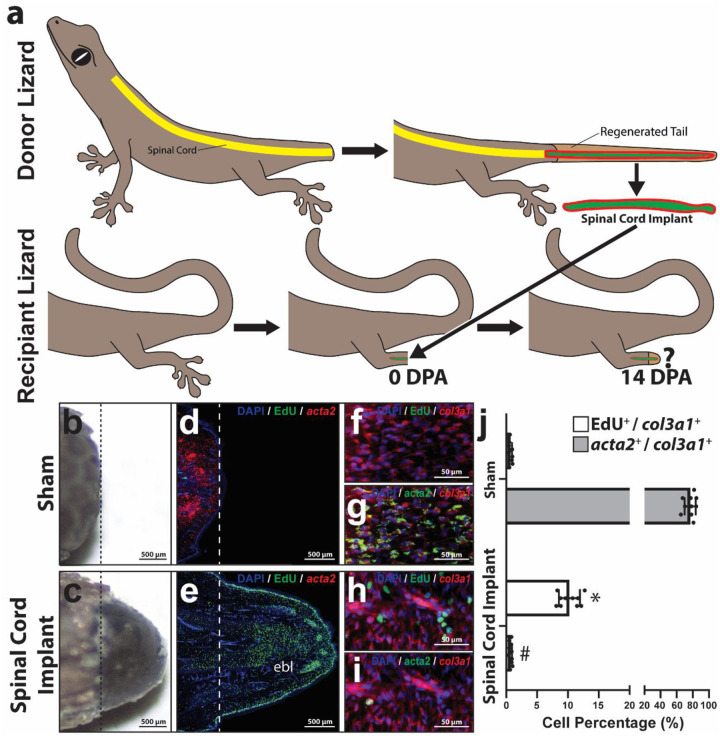
Ectopic blastema induction with lizard spinal ford implants. (a) Experimental scheme used in ectopic limb blastema studies to test effects of tail spinal cord implants on lizard limb healing. Sections of regenerated tail spinal cords were collected from donor lizards 28 DPA and implanted into amputated limbs of recipient lizards. At 14 DPA, limbs were histologically assessed for fibroblast proliferation (EdU imcorpration) and fibrosis (*acta2* FISH). **(b, c)** Gross morphology of representative limbs collected 14 DPA after treatment with (b) sham surgeries (control) or (c) implantation with spinal cord implants. **(d, e)** Sagittal sections collected from lizard limbs treated with sham surgery or spinal cord implants analyzed 14 DPA by FISH/fluorescence microscopy for EdU incorporation and *acta2* expression. **(f-i)** Higher magnification views of distal limb regions analyzed by histology/FISH for EdU incorporation and expression of *col3a1* and *acta2*. **(j)** Quantified percentages of *col3a1*^+^ limb fibroblasts that exhibit EdU incorporation or express *acta2* following treatment with sham surgery or spinal cord implants (14 DPA). n = 10 different animals/tails for each condition. Data are presented as mean values +/− SD. Two-way ANOVA with pairwise Tukey’s multiple comparison tests was used. *, # p < 0.01compared to corresponding sham (control) values.

Having demonstrated the importance of endogenous RGCs and phagocytes in supporting fibroblast proliferation and suppressing fibrosis in naturally regenerating tails, we next sought to determine the effects of introducing these cell populations to lizard limbs, which do not naturally regrow. We also aimed to distinguish the specific roles of microglia from those of other phagocytes in blastema formation. We have previously demonstrated the applicability of the parthenogenetic lizard *Lepidodactylus lugubris* as model organisms for performing tissue transplantation studies without the confounding effects of tissue rejection or immunosuppressive drugs [28, 30, 63, 69]. Spinal cord pieces were isolated from regenerated tails of donor *L. lugubris* lizards pre-treated with AAV5 (GFAPp-GFP) and DiI liposomes as described above and implanted into amputated limb stumps of recipient lizards (Fig. 5 a). Control limbs were treated with sham surgeries that did not involve spinal cord implants. Following 14, and 28 DPA, limbs were analyzed by gross morphology and by histology/FISH for *fabp7*, *sall1*, *col2a1* (cartilage marker [69]), *col3a1*, *acta2*, *mhc* (muscle marker [69]), GFP, and DiI expression (Fig. 5; Supplementary Figure 12, 13). At 14 DPA, spinal cord implants induced ectopic blastema structures on limb stumps involving significant increases in new tissue formation distal to amputation planes compared to sham control limbs, which instead exhibited disorganized scarring (Fig. 5 b–e; Supplementary Figure 14). Like endogenous blastemas, ectopic blastemas were also made up of fibroblasts, and histological analyses indicated that spinal cord implants also significantly increased fibroblast proliferation and significantly reduced fibrocyte differentiation (Fig. 5 f–j).

By 28 days post-limb amputation and spinal cord implantation, ectopic blastemas developed into tail-like growths on limb stumps (Supplementary Figure 12). These “ectopic tails” were patterned similarly to endogenous regenerated tails and formed around central cores of ectopic ependymal tubes that sprouted from spinal cord implants within limb stumps (Supplementary Figure 12, 13). As in endogenous regenerated tails, cartilage tissue surrounded ectopic ependymal tubes, and regenerated muscle formed around cartilage (Supplementary Figure 12 d, f). Like native tail spinal cords, spinal cord implants also contributed to ependymal tube structures within ectopic blastemas/tails (Supplementary Figure 13). Spinal cord implants pre-labeled with GFP^+^ RGCs and DiI^+^ microglia formed ectopic ependymal tubes that extended distal to amputation planes (Supplementary Figure 13 b-d). The presence of GFP^+^ *fabp7*^+^ RGCs and DiI+ *sall1*+ microglia within ectopic ependymal tubes indicates that these populations were reconstituted from spinal cord implant cells. Taken together, these results suggested that lizard limbs contain the necessary response cells, including fibroblasts, to from regenerative structures but lack fibrosis suppression, cell proliferation, and patterning signals provided by spinal cord implants.

### Spinal cord implant induction of ectopic blastemas is dependent upon RGC and microglia populations.

**Figure 6:**
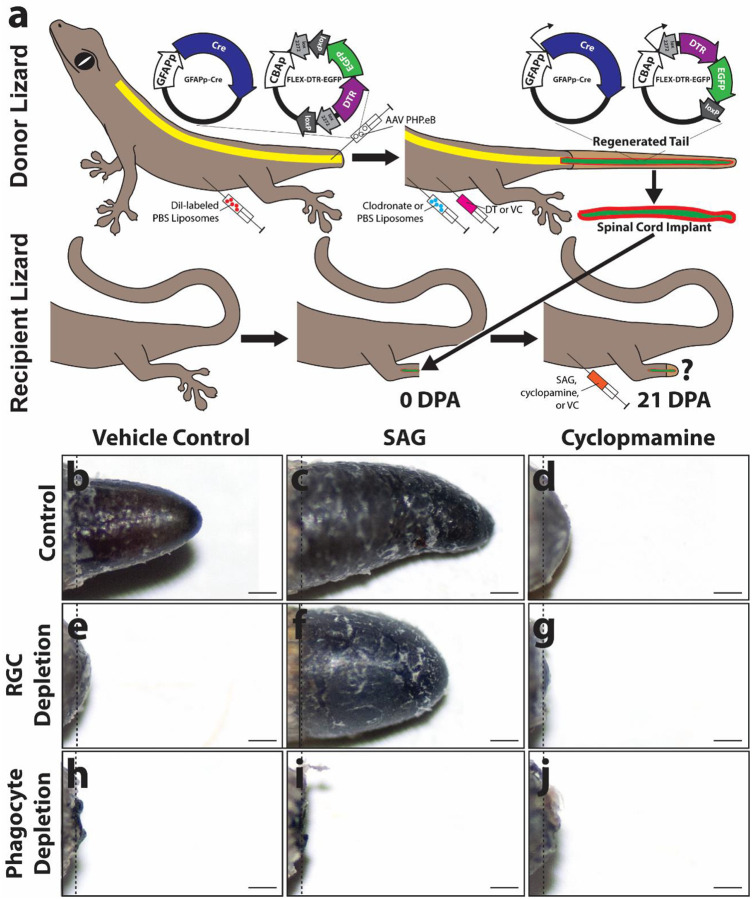
Effects of HH stimulation and inhibition on ectopic blastema induction in amputated lizard limbs treated with spinal cord implants depleted of RGCs or phagocyte populations. **(a)** Experimental scheme used test abilities of SAG and cyclopamine to rescue ectopic limb blastema formation following RGC or phagocyte depletion of spinal cord implants. Spinal cords of donor lizards were pre-transduced with AAV5(GFAPp-Cre) and AAV5(FLEX-DTR-GFP). At 28 DPA, regenerated tail RGCs were depleted by treating with DT, and microglia were depleted by treatment with clodronate liposomes. Pieces of regenerated tail spinal cords were collected from donor lizards and implanted into amputated limbs of recipient lizards. Recipient lizards were treated with SAG, cyclopamine, or vehicle control. At 21 DPA, limbs were collected and analyzed for effects on ectopic blastema formation and limb fibrosis **(b-j)** Gross morphologies of (b-d) limbs treated with control spinal cord implants; (e-g) limbs treated with RGC-depleted spinal cord implants; (h-j) and limbs treated with microglia-depleted spinal cord implants treated with vehicle control, SAG, or cyclopamine (21 DPA). Bar = 500 μm.

Next we tested the effects of RGC and microglia depletion on the abilities of spinal cord implants to induce ectopic blastemas on amputated lizard limbs (Fig. 6). Lizards with amputated tails were pre-treated with AAV5(EGFP/Flex) as previously described (Fig. 6 a). Following 28 days of tail regeneration, donor lizards were treated with DT or clodronate liposomes as described above to deplete RGC or microglia, respectively (Fig. 6 a). Control spinal cord implants, RGC-depleted implants, and microglia-depleted implants were transplanted into amputated lizard limbs. As above, we also sought to test the abilities of HH signaling modulation to rescue effects of RGC and microglia depletion (Fig. 6 a). After 21 DPA, control spinal cord implants induced ectopic blastemas (Fig. 6 b). Treatment with SAG significantly increased ectopic blastema area and length and elevated fibroblast proliferation (Fig. 6 c; Supplementary Figure 15, 16). Cyclopamine treatment inhibited ectopic blastema formation and reduced fibroblast proliferation but did not affect fibrocyte differentiation (Fig. 6 d; Supplementary Figure 15, 16). Similarly, RGC depletion significantly reduced ectopic blastema size, decreased fibroblast proliferation without stimulating fibrosis (Fig. 6 e; Supplementary Figure 15, 16). SAG treatment rescued ectopic blastema formation and fibroblast proliferation following RGC depletion (Fig. 6 f; Supplementary Figure 15, 16). Cyclopamine treatment did not affect fibroblast proliferation or fibrocyte differentiation following RGC depletion (Fig. 6 g; Supplementary Figure 15, 16). Microglia depletion disrupted ectopic blastema formation and reduced fibroblast proliferation but also caused significant increases in fibrocyte differentiation (Fig. 6 h; Supplementary Figure 15, 16). SAG treatment did not rescue ectopic blastema induction or fibroblast proliferation, and neither SAG nor cyclopamine affected ectopic blastema formation or fibrosis suppression following microglia depletion (Fig. 6 i, j; Supplementary Figure 15, 16).

**Figure 7:**
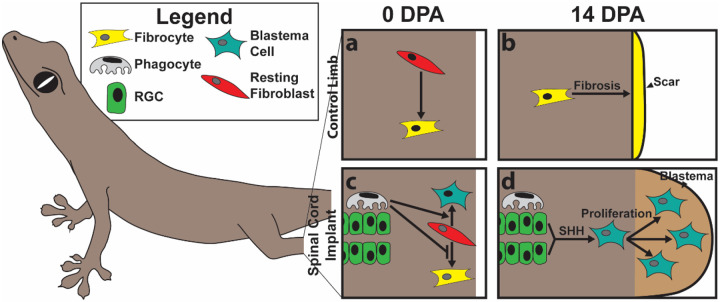
Proposed roles for lizard RGCs and microglia in regulating ectopic blastema formation. **(a)** In amputated control limbs that lack endogenous RGC and microglia populations, fibroblasts differentiate into fibrocytes that **(b)** promote fibrosis and scar formation by 14 DPA. **(c)** In amputated limbs that receive spinal cord implants, exogenous microglia promote fibroblast transition into blastema cells and inhibit fibrocyte differentiation. **(d)** Exogenous RGCs work downstream of microglia activities and provide SHH signals that stimulate blastema fibroblast proliferation and blastema formation by 14 DPA.

Taken together, these results suggest the following mechanisms regulating ectopic lizard blastema development (Fig 7): Amputated lizard limbs do not contain endogenous populations of RGCs or microglia and do not naturally regenerate (Fig. 7 a, b). Upon amputation, limb fibroblasts differentiate into scar-forming fibrocytes (Fig. 7 a, b). Treatment with spinal cord implants introduces exogenous RGC and microglia populations to amputated lizard limbs (Fig. 7 c, d). Microglia promote limb fibroblast differentiation into blastema cells instead of fibrocytes (Fig. 7 c). RGCs produce SHH that stimulate proliferation in blastema cells but not resting fibroblasts or fibrocytes (Fig. 7 d). Scar suppression and blastema cell proliferation leads to ectopic blastema formation (Fig. 7 d).

## DISCUSSION

Our study sheds light on a long-standing question in regenerative biology, “Why can lizards regenerate tails but not limbs?”. Lizards are distinguished as the only adult amniotes that retain blastema-based appendage regeneration capabilities exhibited by amphibians. However, unlike urodeles, which regrow limbs and tails, lizards are only able to regenerate tails [12]. A number of groups including our team have reported that amphibian and lizard blastemas form from proliferating connective tissue fibroblasts [30, 70, 71]. Here we identify spinal cord glia populations as critical signaling sources that direct appendage fibroblasts to form blastemas. This study establishes that lizard limbs contain the necessary starting material (i.e. fibroblasts) to form blastemas but do not naturally regenerate because lizard limbs lack endogenous supplies of redial glia and microglia. Introducing exogenous glia populations in the form of spinal cord implants induces ectopic blastema formation in lizard limbs.

Nerve dependency has been implicated in a wide range of models of wound healing and regeneration, including blastema-based appendage regrowth [16, 20, 25, 39, 40, 72–77]. However, many of these studies were based on denervation procedures and other interventions that involved removal or blockage of whole nerve structures and, therefore, could not distinguish between pro-regenerative effects of nerves and nerve-associated support cells [19, 78]. Indeed, a growing body of evidence suggests that nerve support cells, such as Schwann cells [25, 54, 79] or nerve mesenchyme [55], are at least partially responsible for pro-regenerative activities of nervous tissue. The majority of studies involving nerve-dependent blastema formation have focused on limb regeneration, which are regulated by peripheral nerves that are difficult to physically separate from supporting cells as they invade blastemas. Studies that have focused on adult tail regeneration, which are regulated by spinal cord, not peripheral nerve [12, 28, 50, 56, 63, 80], have focused primarily on salamander tail regeneration. In this respect, studies involving lizard tail regrowth provide interesting context because, unlike those of salamanders, regenerating lizard tails do not naturally regrow spinal cord nerves [28, 63]. Instead, regenerated lizard tail spinal cords take the form of ependymal tubes made up of RGCs, microglia, and fibroblasts [28, 63]. Thus, the regenerated lizard tail model naturally eliminates nerves, leaving only glia and other nerve-support cells to be investigated for their effects on regeneration. Despite these attractive features, lizard models have been understudied in the regenerative sciences compared to the salamander. For example, one of the few prior studies to test the importance of spinal cord tissue in lizard tail regeneration used wax placed over amputation stumps to block ependymal tube invasion [80], a strategy that could not identify the specific ependymal tube cells responsible for regulating blastema formation and patterning. This study overcomes these limitations through selective depletion of specific glial populations. For example, we took advantage of GFAP as a cell type-specific marker within lizard ependymal tubes to target DTR to RGC populations. However, lizard microglia proved more challenging to deplete due to lack of specific markers and phagocytic activity shared with other cell types like macrophage and osteoclasts. Several features of the ectopic blastema model described here facilitated overcoming these hurdles of targeted study of microglia contributions to blastema formation. Our model is made possible by the innovative use of a parthenogenetic lizard species that supports allogeneic cell/tissue transplantations amongst clonally identical offspring without triggering confounding immune reactions and tissue rejections. Sections of lizard ependymal tubes were transferred from donor lizard tails to amputated recipient lizard limbs, which do not naturally contain microglia or form blastemas. Thus, any microglia present within limb tissue were derived from exogenous ependymal tube implants isolated from donor lizards. Furthermore, as the overwhelmingly most abundant phagocyte within lizard ependymal tubes, microglia are specifically depleted via clodronate liposome treatments of donor lizards. Through comparing endogenous cell responses to transplantations of exogenous ependymal tubes collected from control versus clodronate liposome-treated donor lizards, our model supported the direct testing of the specific effects of microglia and microglia depletion on ectopic lizard blastema formation. This strategy of exogenous ependymal tube transplantation also stressed the importance of tissue-resident microglia over blood-derived phagocytes in blastema formation. Indeed, past studies concerning roles of phagocytes in appendage regeneration have focused on cells of the myeloid lineage, including macrophages and osteoclasts [58, 60, 81]. In contrast, this study highlights that microglia and not limb tissue-resident macrophages or blood-derived myeloid cells are the critical phagocytes responsible for fibrosis suppression during lizard blastema formation.

One of the major tools used to demonstrate nerve-dependence in salamander limb regeneration is the accessory limb model [82–84]. This model involves deviating a limb nerve into a lateral wound and grafting skin from the opposing side of the limb axis into the site of injury [85]. The diverted nerve triggers formation of an ectopic limb blastema at the injury site. Ectopic limb blastemas are nearly indistinguishable from those formed in response to amputation injuries and develop into accessory limbs. Here, we essentially describe an accessory tail model capable of forming ectopic tail blastemas that develop identically to endogenous regenerated tails. However, several key differences distinguish our accessory lizard tail model from the salamander limb model; instead of diverting a nerve to another position on the same appendage and animal, our model involves the transplantation of regenerated spinal cord tissue collected from donor lizard tails into recipient lizard limbs. Combined with the dichotomy of lizard appendage regenerative capacities, our model affords the attractive opportunity to test the effects of introducing tissue from regenerative tails into non-regenerative limbs. As described above, our accessory tail model also allows for the manipulation of specific spinal cord cell populations prior to transplantation towards better characterizing their roles in ectopic blastema formation. Specifically, this strategy provides options for transgene introduction and labeling that are especially important in overcoming the hurdles that come with the current lack of transgenic lizards. Realities of lizards reproduction make organismal transgenesis difficult, and introduction of exogenous genes like Cre, GFP, and DTR used in our study remain beyond current capabilities [86]. The experimental scheme described here incorporates several strategies for addressing these challenges. For example, we show that the combined use of AAV5 vectors and a conserved GFAP promoter are effective for targeting transgene expression to RGCs.

This study identified distinct roles for lizard spinal cord microglia and radial glia populations in regulating lizard blastema formation. Radial glia support blastema cell proliferation directly via stimulation of HH signaling, effects that can be largely rescued via treatment with HH agonists. Conversely, microglia act upstream of radial glia and enhance blastema cell responsiveness to HH stimulation. We also uncovered roles for microglia, but not RGCs, in suppressing fibrosis following appendage amputation. We have previously identified enhanced HH responsivity as a specific hallmark of the lizard blastema cell state during derivation from tail wound fibroblasts [30, 56, 57, 68]. We have also showed that blastema cells and fibrocytes derive from identical starting fibroblast populations [30, 57]. The data presented here suggest that microglia both support enhanced fibroblast HH responsiveness and suppress fibrocyte differentiation, and we suspect that these characteristics are related. Specifically, we hypothesize that microglia support blastema cell derivation via epigenetic enhancement of chromatin accessibilities within HH-responsive genetic elements. It is well established that stimulation via HH signaling results in activation of the GLI family of transcription factors [87, 88]. Activated GLI transcription factors bind accessible Hedgehog response elements (HHREs) within promoters/enhancers containing the recognition sequence GACCACCCA, resulting in expression of corresponding genes. Many genes regulating cell proliferation and differentiation, including SULF1 and SOX9, contain HHRE within their promoters/enhancers [89], but epigenetic regulation via DNA methylation, histone binding, etc. limits HHRE accessibilities to GLI binding and, subsequently, transcription. We hypothesize that lizard spinal cord microglia potentiate signaling that reprograms wound fibroblasts to a blastema cell state by increasing genetic accessibilities of HHREs to GLI1 binding in the promoters/enhancers of proliferation genes. While beyond the scope of this current paper, identifying the fibroblast signaling pathways and specific HHREs under microglia regulation will be the subject of future studies.

Finally, these results and model provide interesting context for comparisons with mammalian wound healing models. For example, in mouse spinal cord injuries, glial cells contribute to disorganized scars that physically block healing [90, 91], and microglia are reported to hinder spinal cord regeneration [92]. Here we report the opposite for lizard RGCs and microglia; lizard RGCs self-organize and regenerate ependymal tubes that coordinate blastema formation. Lizard microglia direct tail and limb fibroblast responses to injury away from fibrosis and toward blastema establishment. These results suggest fundamental differences between mammalian and lizard glia populations and their respective responses to injury signals that may be subjects for future study. Furthermore, these points are particularly interesting given how similar mammalian and lizard fibroblast populations are to one another, and it is interesting to speculate that mammalian limbs may be under similar regenerative constraints as those of lizards. Perhaps mammalian limb regeneration can be induced using a similar scheme of introducing signaling centers that fill the same roles of lizard RGCs and microglia toward suppressing fibrosis and supporting cell proliferation and new tissue growth.

A limitation of this study is that true limb regeneration was not achieved. While we did succeed in suppressing fibrosis and stimulating blastema formation in appendages not naturally capable of regeneration, the ectopic blastemas that did form were decidedly lizard tail, and not limb, structures. Indeed, when allowed to develop further, ectopic blastemas developed into tail-like structures that exhibited hallmarks specific to regenerated lizard tails, including unsegmented cartilage tubes and ependymal tubes. Thus, these studies support the principle that not all blastemas are equal; instead, appendage-specific differences in signaling centers regulating blastema differentiation and patterning result in divergent final structures. Since our ectopic blastema model involved tail-specific signaling centers, i.e. ependymal tubes, amputated limbs regenerated tail-like growths instead of limbs. Future work will be aimed at identifying changes that can be made to the presented ectopic blastema model toward improving regeneration fidelities and reforming limb structures rather than tail. For example, we have previously used embryonic cell transplantations to manipulate tissue patterning and improve regeneration fidelities during adult lizard appendage regrowth in *L. lugubris*. Perhaps a similar strategy involving a combination of spinal cord microglia, to suppress fibrosis, in conjunction with signaling centers from embryonic limb development, such as zone of polarizing activity and apical ectodermal cap, would support limb-specific patterning of ectopic blastemas and reform a limb instead of a tail.

In summary, this study demonstrates the importance of spinal cord RGC and microglia populations in lizard blastema formation. Results involving microglia, in particular, bridge previous separate reports on the critical roles of phagocytes and nervous tissues in appendage regeneration and fibrosis suppression. This work was made possible through the development of an ectopic blastema model that effectively induces tail-like growths on amputated lizard limbs following implantation of tail ependymal tube segments. Lizards are the closest relatives of mammals, and the only amniotes, capable of multi-lineage, blastema-based appendage regrowth, and the ectopic limb blastemas described here are distinguished as the only such regenerative structures to be induced on amputated amniotes limbs to date. Such distinctions highlight the particular relevancy to these findings towards benefitting human patients, and this study provides proof-of-concept for suppressing scarring and supporting new tissue growth in limbs not naturally capable of regrowth. Recreating even the earliest stages of lizard blastema formation holds promise to limit painful scarring, reduce dysregulated ossification, and support new organized tissue formation. Any one of these milestones would facilitate prosthesis attachment and introduce much-needed improvements to patient quality of life following amputation injuries.
